# The genetic basis of differential autodiploidization in evolving yeast populations

**DOI:** 10.1093/g3journal/jkab192

**Published:** 2021-06-02

**Authors:** Sudipta Tung, Christopher W Bakerlee, Angela M Phillips, Alex N Nguyen Ba, Michael M Desai

**Affiliations:** 1 Department of Organismic and Evolutionary Biology, Harvard University, Cambridge, MA 02138, USA; 2 The Lakshmi Mittal And Family South Asia Institute, Harvard University, Cambridge, MA 02138, USA; 3 Quantitative Biology Initiative, Harvard University, Cambridge, MA 02138, USA; 4 NSF-Simons Center for Mathematical and Statistical Analysis of Biology, Harvard University, Cambridge, MA 02138, USA; 5 Department of Molecular and Cellular Biology, Harvard University, Cambridge, MA 02138, USA; 6 Department of Physics, Harvard University, Cambridge, MA 02138, USA

**Keywords:** experimental evolution, *Saccharomyces cerevisiae*, whole-genome duplication, ploidy evolution, QTL analysis

## Abstract

Spontaneous whole-genome duplication, or autodiploidization, is a common route to adaptation in experimental evolution of haploid budding yeast populations. The rate at which autodiploids fix in these populations appears to vary across strain backgrounds, but the genetic basis of these differences remains poorly characterized. Here, we show that the frequency of autodiploidization differs dramatically between two closely related laboratory strains of *Saccharomyces cerevisiae*, BY4741 and W303. To investigate the genetic basis of this difference, we crossed these strains to generate hundreds of unique F1 segregants and tested the tendency of each segregant to autodiplodize across hundreds of generations of laboratory evolution. We find that variants in the *SSD1* gene are the primary genetic determinant of differences in autodiploidization. We then used multiple laboratory and wild strains of *S. cerevisiae* to show that clonal populations of strains with a functional copy of *SSD1* autodiploidize more frequently in evolution experiments, while knocking out this gene or replacing it with the W303 allele reduces autodiploidization propensity across all genetic backgrounds tested. These results suggest a potential strategy for modifying rates of spontaneous whole-genome duplications in laboratory evolution experiments in haploid budding yeast. They may also have relevance to other settings in which eukaryotic genome stability plays an important role, such as biomanufacturing and the treatment of pathogenic fungal diseases and cancers.

## Introduction

As populations evolve, they occasionally undergo changes in ploidy. These changes have led to extensive ploidy variation across the tree of life, including notable differences among fungi (Albertin and Marullo 2012), plants, animals, and other eukaryotes (reviewed in Otto 2007; Sémon and Wolfe 2007). Ploidy changes and broader genome instability have also been observed in clinically relevant contexts, where they appear to contribute to fungal pathogenesis (Morrow and Fraser 2013) and tumorigenesis (Fujiwara *et al.* 2005; Storchova and Kuffer 2008).

In several recent laboratory evolution experiments with *Saccharomyces cerevisiae*, populations have been found to spontaneously duplicate their whole genomes, or autodiploidize, with high frequency in the early stages of adaptation (Gerstein *et al.* 2006; Oud *et al.* 2013; Hong and Gresham 2014; Levy *et al.* 2015; Voordeckers *et al.* 2015; Gorter *et al.* 2017; Fisher *et al.* 2018; Kosheleva and Desai 2018; Nguyen Ba *et al.* 2019). In one such experiment, autodiploidization events were found to have a substantial fitness benefit, and make up the vast majority of initial beneficial mutations (Venkataram *et al.* 2016). Autodiploidization occurs in these strains despite mutations at the homothallic switching endonuclease (*HO*) locus that sharply reduce the frequency of mating-type switching (Haber *et al.* 1980).

While some work has been done to illuminate how different environmental conditions affect the propensity for autodiploids to arise and increase to appreciable frequency (Harari *et al.* 2018), the genetic basis of this trait remains uncharacterized. This leaves a significant gap in our understanding of perhaps the most commonly observed mutation in yeast laboratory evolution experiments. This gap also presents a practical challenge for researchers conducting yeast evolution experiments, where autodiploidization frustrates efforts to study the evolutionary consequences of ploidy-dependent population genetic parameters, including mutation rates, recombination, and the distribution of fitness effects. In addition, autodiploidization can complicate efforts to genetically manipulate budding yeast, such as by adding DNA barcodes (Levy *et al.* 2015) or activating more complex genetic circuitry [*e.g.*, Cre-*lox* recombination machinery (Nguyen Ba *et al.* 2019)], especially in the context of long-term culture. Thus, a better understanding of the genetic basis of this trait may benefit both researchers in experimental evolution and those who use or study yeast in industry, medicine, and molecular biology.

Previous evolution experiments founded with haploid clones derived from budding yeast strains BY4741 and W303 have suggested that BY-derived populations fix autodiploids more frequently than W303-derived populations (*e.g.*, Hong and Gresham 2014; Levy *et al.* 2015; Voordeckers *et al.* 2015; Gorter *et al.* 2017; for BY; Jerison *et al.* 2020; Johnson *et al.* 2021; for W303, but see Fisher *et al.* 2018). Here, we combine experimental evolution with a QTL mapping approach to identify the genetic basis for this difference in propensity to autodiploidize. Consistent with recent work describing the genetic basis for aneuploidy tolerance in wild yeast (Hose *et al.* 2020, although see Scopel *et al.* 2021) we identified alleles of the *SSD1* gene as the primary genetic determinant of this difference. Below, we describe the experiments that led to this finding and its confirmation, and we speculate briefly about the underlying biological mechanism.

## Materials and methods

### Yeast strains and F1 segregants for QTL mapping

**Figure 1:**
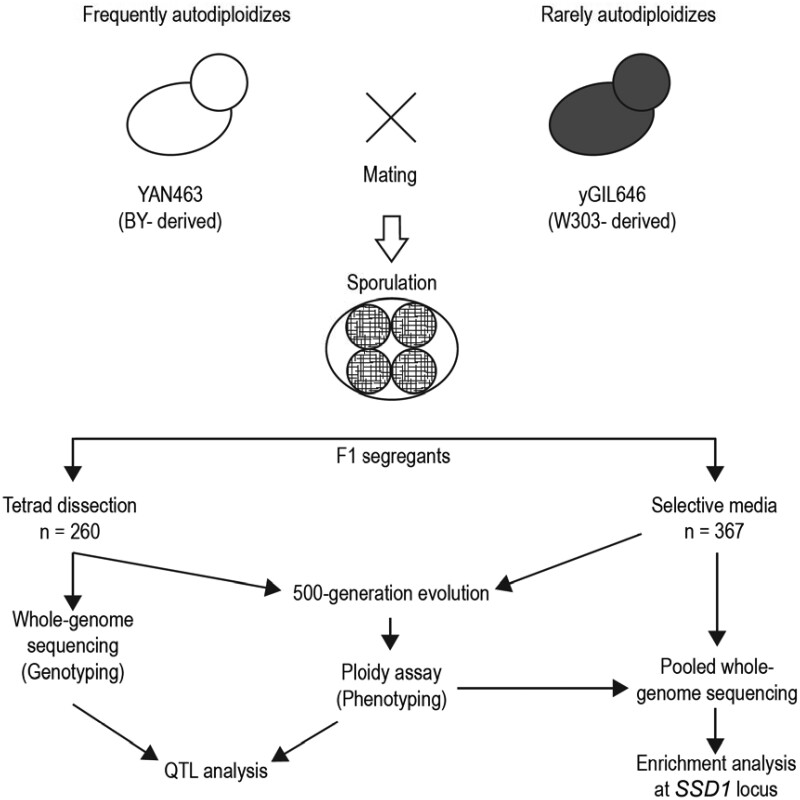
Schematic diagram of the QTL mapping experiment. Parents with different autodiploidization propensities were crossed, and F1 segregants either dissected from tetrads (“tetrad spores”) or selected in bulk on selective media (“selected spores”). All spores were subject to 500 generations of evolution in rich media. At the conclusion of the evolution experiment, the ploidy of all populations was assayed via flow cytometry. All “tetrad spores” were genotyped individually via whole-genome sequencing, and the combined genetic and phenotypic data were used to detect QTLs. The “selected spores” were sequenced in pools and analyzed for enrichment of the identified QTL, *SSD1*.

To generate F1 segregants for QTL mapping, we used BY-derived YAN463 (*MAT***a**, *his3*Δ*1*, *ura3*Δ*0*, *leu2*Δ*0*, *lys2*Δ*0*, *RME1pr::ins-308A*, *ycr043c*Δ*0::NatMX*, *ybr209w::CORE-UK*, *can1::STE2pr_SpHIS5_STE3pr_LEU2*) as the parent that frequently autodiploidized, while W303-derived yGIL646 (*MAT*α, *ade2-1*, *CAN1*, *his3-11*, *leu2-3,112*, *trp1-1*, *bar1*Δ*::ADE2*, *hmlα*Δ*::LEU2*, *GPA1::NatMX*, *ura3*Δ*::PFUS1-yEVenus*), described elsewhere (Fisher *et al.* 2018), served as the parent that rarely autodiploidized (Figure 1). Note that we included the *RME1pr::ins-308A* mutation in our BY strain to increase its sporulation efficiency. The *CORE-UK* cassette was originally included to facilitate knocking new genetic material into the *YBR209W* locus via the *delitto perfetto* method (Storici and Resnick 2006), but it was incidental to this study. After mating and sporulation, we isolated a total of 627 haploid F1 offspring (segregants), in three separate sets. The first set of segregants was constructed by dissecting 65 tetrads, yielding 260 “tetrad spores.” The second and third sets consisted of 184 and 183 *MAT***a** F1 segregants respectively, each set with common auxotrophies, which we isolated by germinating spores on synthetic defined (SD) growth medium with canavanine but without adenine, histidine (SD –Ade –His +Can), and without adenine, histidine, uracil, tryptophan (SD –Ade –His –Ura –Trp +Can), respectively. Note that since the W303 strain was auxotrophic for histidine and BY’s *Schizosaccharomyces pombe*-derived *HIS5* (orthologous to *S. cerevisiae’*s *HIS3)* was under control of the *MAT***a**-specific *STE2* promoter, we were able to select for *MAT***a** spores by excluding histidine from the selection media. We refer to these segregant sets as “selected spores” hereafter.

### Experimental evolution

To assess autodiploidization propensity, we founded seven replicate populations from individual clones of each of the two parental genotypes, and one replicate population from each of the 627 F1 segregants. We propagated each of the resulting 641 populations for 500 generations in unshaken flat-bottom polypropylene 96-well microplates using a standard batch culture protocol (with 1:2^10^ dilutions every 24 hours). All evolution was conducted at 30°C in 128 µl of YPD [a rich laboratory media; 1% Bacto yeast extract (VWR #90000-722), 2% Bacto peptone (VWR #90000-368), 2% dextrose (VWR #90000–904)] with ampicillin sodium salt [100 µg/mL (VWR #97061-440)]. All liquid handling was conducted using a BiomekFX^P^ robot (Beckman Coulter), as described previously (see *e.g.*, Lang *et al.* 2011). To detect contamination and cross-contamination events, each 96-well plate contained a unique pattern of “blank” wells containing only media. No contamination was observed in the blank wells at any point during this experiment. At 50-generation intervals, we froze aliquots of all populations in 10% glycerol at −80°C. Prior to conducting ploidy assays and sequencing library preparation, we revived the relevant populations by thawing and inoculating 4 µl of each into 124 µl YPD at 30°C.

### Examining ploidy by nucleic acid staining

After evolving for 500 generations, we evaluated the ploidy status of each population by staining for DNA content using a procedure previously described (Jerison *et al.* 2020; Johnson *et al.* 2021), with slight modifications. Briefly, 6 µl of saturated culture from each population was added to 120 µl water in a fresh 96-well plate and centrifuged (2000 rcf, 2 minutes). To fix the cells, supernatants were removed, and the pellets were resuspended by gentle pipetting in 150 µl of 70% ethanol and incubated for 1 hour at room temperature. The samples were then centrifuged (2000 rcf, 2 minutes), supernatants were removed, and cells were resuspended in 65 µl RNAase A solution consisting of 10 mg/mL RNAase A (VWR Life Science, 9001-99-4) dissolved in 10 mM Tris-HCl, pH 8.0 and 15 mM NaCl, and incubated for ∼4 hours at 37°C. Subsequently, 65 µl of 2 µM SYTOX green (Thermo Fisher Scientific, S7020) in 10 mM Tris-HCl, pH 8.0 was added to each sample, shaken briefly on a Titramax 100 plate shaker (Heidolph Instruments) for approximately 30 seconds, and incubated in the dark for at least 20 minutes at room temperature. The samples were then analyzed using a Fortessa flow cytometer (BD Biosciences). DNA content of ∼10,000 cells of each sample was measured through a linear FITC channel and, using Flowing software version 2.5.1 (Turku Bioscience), FITC histograms (Supplementary Figures S1 and S2) were compared to known haploid and diploid controls to identify their ploidy.

### Genotyping with whole-genome sequencing

We genotyped all 260 F1 segregants from the tetrad spore set using whole-genome Illumina sequencing at ∼5X coverage, and the parental strains YAN463 and yGIL646 at 125X and 40X coverage, respectively. To account for parental differences in auxotrophies at lysine and tryptophan, which we suspected might affect autodiploidization propensity, we grouped “selected spores” based on their lysine and tryptophan auxotrophy and ploidy status after evolution and sequenced the eight resulting pooled samples (Lys proto-/auxotrophy × Trp proto-/auxotrophy × haploid/autodiploidized).

To prepare sequencing libraries for all samples in parallel, we used a BiomekFX^P^ liquid handling robot (Beckman Coulter) to extract total genomic DNA from ∼500 µl saturated cultures of all samples, following a previously described procedure (Johnson *et al.* 2021). A high-throughput Bio-On-Magnetic-Beads (BOMB) protocol with paramagnetic beads and GITC lysis buffer (Oberacker *et al.* 2019) was used for this step, followed by DNA quantification using the AccuGreen™ High Sensitivity dsDNA Quantitation kit (Biotium, 31066) on clear flat-bottom 96-well polystyrene plates (Corning^®^, VWR Life Science, 25381-056). Extracted genomic DNA was then subjected to Nextera tagmentation (following Baym *et al.* 2015) in preparation for multiplexed Illumina sequencing. Tagmented PCR products were then purified using PCRcleanDX magnetic beads (Aline Biosciences) through a two-sided size selection procedure with 0.5/0.75X or 0.5/0.8X bead buffer ratios (Johnson *et al.* 2021). Quality of the multiplexed libraries was verified by estimating their fragment-size distributions using the Agilent 4200 TapeStation system and sequenced with 2 × 150 bp paired-end chemistry on Illumina NextSeq 500 and Illumina NovaSeq platforms.

After obtaining raw sequence reads, we first trimmed them using Trimmomatic v0.36 (Bolger *et al.* 2014). We then aimed to obtain parental reference genomes and construct a list of the single nucleotide polymorphisms (SNPs) that are different between them. First, we subjected the reads for the BY-derived parent, YAN463, to a Breseq v0.31.0 pipeline (Deatherage and Barrick 2014) with BY4742 genome assembly reference sequence (GCA_003086655.1) in order to identify variants. Using Breseq’s gdtools utility program, the identified variants were applied back into the BY4742 reference genome to create an updated BY-parental genome reference. Next, the reads for the W303-derived parent, yGIL646, were parsed through Breseq v0.31.0 pipeline using the newly constructed updated BY-parental genome as a reference. The identified SNPs were incorporated into the updated BY-parental genome reference using Breseq’s gdtools utility program to construct an updated W303-parental genome reference. This ensured that the location of each SNP is identical in both parental genome references. The parental genome references were then compared to identify a list of 8505 SNPs, differing between these two genome references. Subsequently, this list of SNPs was used to identify from which parent (BY or W303) each locus was inherited in all the tetrad spores. In short, sequences for each tetrad segregant were checked for appropriate coverage and quality, the reads were aligned to BY- and W303-parental reference genome sequence separately using bowtie2 and indexed using samtools. We identified the number of reads matching each parental reference at each locus using Python and inferred genotype at each of these loci using a hidden Markov model (HMM) algorithm. Sequences for two segregants were disregarded due to insufficient read count.

Similarly, for the eight pooled samples of the selected spores, the number of sequencing reads matching BY and W303 parental sequences at each of the 8505 loci were computed. These data were used for the enrichment analysis described below.

### QTL mapping

Our dataset consisting of genotypes (B or W, corresponding to the BY and W303 parental background respectively) at 8505 loci (columns) of 258 segregants (rows) and their ploidy phenotype after evolution (binary data, haploid = 0, diploid = 1) was used as the input for QTL analysis using R/qtl v1.46-2 software as described below (following Broman and Sen 2009). Before QTL mapping, a battery of diagnostic probes, involving a test for segregation distortion of the markers and an analysis of anomalous genotyping similarity and number of crossover events for the segregants, were checked to avoid spurious mapping (see Supplementary Text S1 for details). This resulted in a clean dataset consisting of genotypes of 255 segregants at 8475 loci with their corresponding phenotypes, which then entered the following QTL mapping pipeline.

First, we computed LOD scores for all 8475 loci assuming the presence of a single QTL using standard interval mapping and the Haley–Knott regression method for a binary phenotype with LOD significance thresholds computed from 10000 permutations. Next, to find any potential interactions between multiple QTLs, we divided our data into predictor and test datasets. We chose 150 segregants arbitrarily to form a predictor dataset and subjected their genotype and phenotype data to a forward/backward stepwise search algorithm (stepwiseqtl) with LOD significance thresholds computed from 1000 permutations. Based on the LOD score profile of single-QTL analysis above (see Results) we restricted this search to chromosomes IV and XIV only, and the maximum number of QTLs allowed in a model was kept to 4. Subsequently, we fitted the predicted QTL model onto the remaining data consisting of 105 segregants (test dataset) using fitqtl followed by the refineqtl function.

Furthermore, to reveal any additional low-effect QTL for the autodiploidization phenotype, we rescanned the data using single-QTL analysis methods after regressing out the QTL with highest LOD score obtained above. Effect sizes of the two alleles of the QTL with statistically significant LOD score were estimated using the effectplot function.

### Enrichment analysis

For each of the eight pooled samples (Lys proto-/auxotrophy × Trp proto-/auxotrophy × haploid/autodiploidized) of “selected spores,” we scanned their sequencing reads at the SNP that led to statistical significance in the QTL analysis above. The proportion of those reads matching with BY version of the QTL locus was computed to find whether this statistic was different in the haploid and diploid pool.

### Experimental validation of QTL mapping result

To validate the results of our QTL mapping analysis, we cleanly knocked out the entire open reading frame (ORF) of the gene containing the statistically significant QTL, *SSD1*, using a *HygMX* or *KanMX* cassette in BY4741, W303-derived yGIL104, and RM-derived YAN516 (Table 1). Our *HygMX* cassette, which conferred resistance to hygromycin, was under *Ashbya gossypii TEF1* promotion and termination (Wach *et al.* 1994). Our *KanMX* cassette, which conferred resistance to G418, was under control of the *TEF1* promoter from *Kluyveromyces lactis* and under tSynth8 termination (Curran *et al.* 2015). The *KanMX*-constructed strains were used in the subsequent lab evolution experiment.

**Table 1:** List of experimental strains used and their genotypes

Strain name/ID	Genotype	Reference
QTL mapping		
yGIL646	*MAT* **α**, *ade2-1*, *CAN1*, *his3-11*, *leu2-3,112*, *trp1-1*, *bar1Δ::ADE2*, *hmlαΔ::LEU2*, *GPA1::NatMX*, *ura3Δ::PFUS1-yEVenus*	Fisher *et al.* (2018)
YAN463	*MAT* **a**, *his3Δ1*, *ura3Δ0*, *leu2Δ0*, *lys2Δ0*, *RME1pr::ins-308A*, *ycr043cΔ0::NatMX*, *ybr209w::CORE-UK*, *can1::STE2pr_SpHIS5_STE3pr_LEU2*	This study; Storici and Resnick (2006) (CORE-UK)
Empirical validation of QTL mapping result		
BY4741	*MAT* **a**, *his3Δ1*, *ura3Δ0*, *leu2Δ0*, *met17Δ0*	Brachmann *et al.* (1998)
yGIL104	*MAT* **a**, *URA3*, *leu2*, *trp1*, *CAN1*, *ade2*, *his3*, *bar1Δ::ADE2*	Lang and Murray (2008)
YAN516 (RM)	*MAT* **a**, *ura3Δ0*, *leu2Δ0*, *his3Δ1*, *AMN1*(A1103T), *HO::KwpTEF-NAT-tSynth7*	Brem *et al.* (2002)
yGL0005 (Y55)	*lys2Δ hoΔ*::*LYS2*; Created from yGL0006 (NKY177) by tetrad dissection, selection on LYS- and MT test	Courtesy of Gal Lumbroso
YCB168A/B, YCB172A/B, YCB173A/B	BY4741: *ssd1::KlpTEF-KanMX-tSynth8*	This study
	(*i.e.*, BY4741, *ssd1*Δ)	
YCB169A/B, YCB174A/B, YCB175A/B	yGIL104: *ssd1-d::KlpTEF-KanMX-tSynth8*	This study
	(*i.e.*, yGIL104, *ssd1-d*Δ)	
YCB170A/B	YAN516: *ssd1::KlpTEF-KanMX-tSynth8*	This study
	(*i.e.*, YAN516, *ssd1*Δ)	
YCB176A/B, YCB177A/B	BY4741: *ssd1::SSD1-tGuo1_KlpTEF-KanMX-tSynth8*	This study
	(*i.e.*, BY4741, *ssd1Δ::SSD1*)	
YCB178A/B, YCB179A/B	yGIL104: *ssd1-d::SSD1-tGuo1_KlpTEF-KanMX-tSynth8*	This study
	(*i.e.*, yGIL104, *ssd1-dΔ::SSD1*)	
YCB180A/B, YCB181A/B	BY4741: *ssd1::ssd1-d-tGuo1_KlpTEF-KanMX-tSynth8*	This study
	(*i.e.*, BY4741, *ssd1Δ::ssd1-d*)	
YCB182A/B, YCB183A/B	yGIL104: *ssd1-d::ssd1-d-tGuo1_KlpTEF-KanMX-tSynth8*	This study
	(*i.e.*, yGIL104, *ssd1-dΔ::ssd1-d*)	
YAN500 (SK1)	*MAT* **a**, *his3Δ200, lys2, leu2, trp1, ura3*	Conrad *et al.* (1997), Courtesy of Katya Kosheleva
YPS128	*ura3::KanMX, ho::HygMX*	SGRP (Cubillos *et al.* 2009)
DBVPG1106	*ura3::KanMX, ho::HygMX*	SGRP (Cubillos *et al.* 2009)

Furthermore, starting with the BY and W303 strains in which *HygMX* replaced the *SSD1* ORF, we (re-)integrated the BY and W303 *SSD1* alleles alongside *KanMX*, as described above. The *SSD1* alleles were placed under the strains’ native *SSD1* promoters and terminated by tGuo1, just upstream of *KanMX* (Curran *et al.* 2015). This produced versions of BY4741 and W303 in which either the BY or W303 *SSD1* allele was present at the *SSD1* locus (*i.e.*, four strains total). As a control, in the BY and W303 strains in which *HygMX* was used to knock out *SSD1*, we replaced *HygMX* with *KanMX*, producing a set of *KanMX*-based *SSD1* knockouts ostensibly identical to those described above.

Yeast transformations for strain construction were conducted as described by Gietz (2015), introducing new genetic material as PCR amplicons for incorporation by homologous recombination. A list of the primers used is provided in Table 2. Colony PCR and Sanger sequencing were used to confirm the proper integration of amplicons. During strain construction, independent transformant colonies were picked at each step to produce biological replicates and mitigate the phenotypic effects of any unintended off-target mutations. Sytox staining and flow cytometry were used to verify that all ancestral strains were indeed haploid.

**Table 2:** List of primers used in strain construction

Name	Sequence	Description
pSSD1>pTEF-F	TTC AGC GCA AAG ATT TGG CCC AAT TAT TCC ATC TTT ATA CAC TAG CTT GCC TCG TCC CCG	To amplify *HygMX* for initial *SSD1* knockout
tSSD1>tTEF-R	AAA AAC AAG AAA AAC AGC AAT GAC GAT ATT GGT AGA AGA GAT GGA TGG CGG CGT TAG TAT	To amplify *HygMX* for initial *SSD1* knockout
pSSD1>KlpTEF-F	GCG CAA AGA TTT GGC CCA ATT ATT CCA TCT TTA TAC ACT AAC ACT GGG TCA ATC ATA GCC	To amplify *KlpTEF-KanMX-tSynth8* for *SSD1* knockout
tSSD1>tSynth8-R	AAA AAC AGC AAT GAC GAT ATT GGT AGA AGA GAT TTG AAA GAT GAT ACT CTT TAT TCC TAC	To amplify *KlpTEF-KanMX-tSynth8* for *SSD1* knockout, knock-ins
SSD1-upstream-F	AGC TGA GAA ATA GGA GAG ATT ATA TTT TAG	To amplify *SSD1* alleles for knock-ins
tGuo1>SSD1-R	TGA AAG ATG ATA CTC TTT ATT TCT AGA CAG TTA TAT ATT ATA CCC TCT TCA TGA ATG GAT	To amplify *SSD1* alleles for knock-ins
tGuo1>KlpTEF-F	TAT ATA ACT GTC TAG AAA TAA AGA GTA TCA TCT TTC AAA AAC ACT GGG TCA ATC ATA GCC	To amplify *KlpTEF-KanMX-tSynth8* for *SSD1* allele knock-ins

We compared the tendency for populations founded with these strains to autodiploidize with each other and with corresponding parental controls by clonally propagating them for 500 generations alongside parental controls and examining their ploidy status after evolution by Sytox staining and flow cytometry. There were 22 technical replicates for each strain construct except for BY4741, *ssd1*Δ and yGIL104, *ssd1-d*Δ, which had 44 technical replicates each. One well for yGIL104, *ssd1-d*Δ*::SSD1* was contaminated by bacteria and thereafter removed. Technical replicates of each genotype were split among at least two biological replicates of that genotype. Populations were frozen initially and at 50-generation intervals in 8% glycerol.

In addition, we investigated autodiploidization propensity of two domesticated (SK1 and Y55) and two wild *S. cerevisiae* strains (YPS128 and DBVPG1106) following 500 generations of evolution, using a similar approach to the above with at least 12 technical replicates each. All these strains harbor a functional *SSD1* gene. A consolidated list of all the strains and their genotypes used in this study is provided in Table 1.

### Data availability

All the strains used here are available from the corresponding author upon request. Raw DNA sequencing reads have been deposited in the NCBI BioProject database with accession number PRJNA713332. Additional information regarding strains whose sequences have been uploaded to NCBI can be found in Supplementary File S1. Data used for all the figures are available in Supplementary File S2. Supplementary material is available at figshare: https://doi.org/10.25387/g3.14696256.

## Results

### Autodiploidization propensity differs across two closely related laboratory strains of *S. cerevisiae*

**Figure 2:**
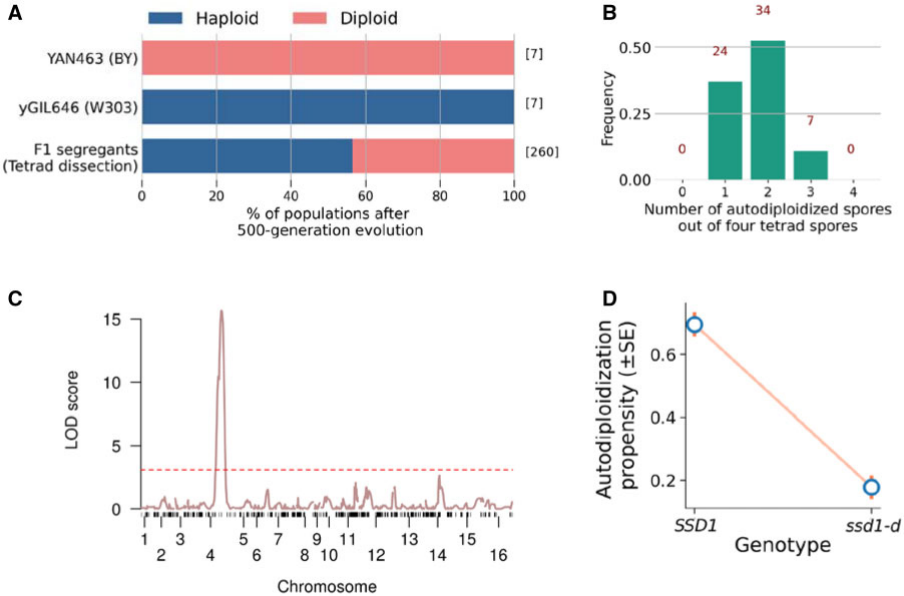
QTL mapping identified a single locus driving variation in autodiploidization propensity. (A) Percentage of populations autodiploidized among the clonal replicates of the two parental strains (YAN463 and yGIL646) and their F1 segregants (tetrad spores) after evolving for 500 generations. The numbers inside square brackets denote the number of populations in each category. (B) Histogram of the number of autodiploidized spores out of four spores in a tetrad. The numbers in red denote the number of tetrads in each category. (C) LOD score for variation in autodiploidization is plotted against the genetic map. The red dashed line indicates a 5% LOD significance threshold computed from 10,000 permutations. The one statistically significant QTL contains a single SNP in the *SSD1* gene. (D) Autodiploidization propensity conditional on BY (*SSD1*) and W303 (*ssd1-d*) alleles respectively across all tetrad spores.

To investigate the intrinsic difference in autodiploidization between BY and W303 populations, we founded seven populations from single clones of each the BY-derived YAN463 and W303-derived yGIL646, respectively, and evolved these for 500 generations in rich media. After evolution, we found that all seven replicate YAN463 populations and none of the yGIL646 populations fixed autodiploids (Figure 2A and Supplementary Figure S1). We also found that seven replicate populations founded by the specific ancestral isolate of yGIL646 used in Fisher *et al.* (2018) also failed to fix autodiploids during 500 generations of evolution.

In parallel, we conducted a QTL evolution experiment (Figure 1). We first crossed and sporulated yGIL646 and YAN463, dissecting 65 tetrads to obtain 260 F1 segregants. We then founded one population from each of these segregants, and evolved in rich media at 30°C in 96-well plates for 500 generations. Close to half of these populations autodiploidized within 500 generations (44%, 113 out of 260 spores; Figure 2A). In 52% of the tetrads (34/65), two out of four spore-derived populations diploidized while the other two remained haploid. In 37 and 11% of the tetrads, one and three spore-derived populations diploidized, respectively. In none of these tetrads did all four spore-derived populations autodiploidize or remain haploid (Figure 2B).

**Figure 3:**
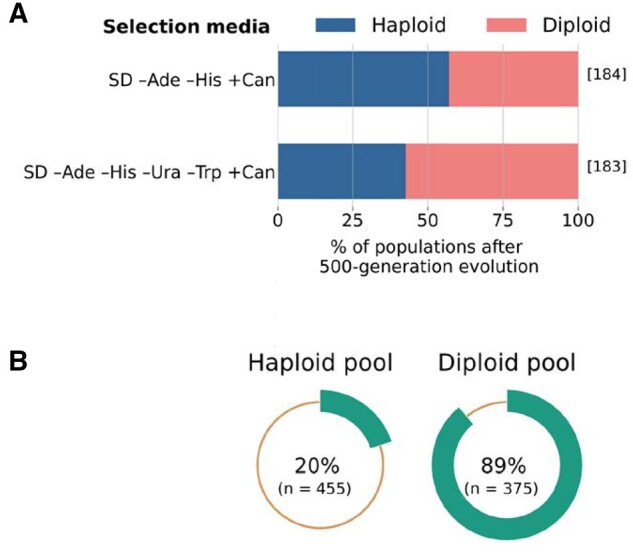
Ploidy status of the “selected spores” after evolution, and enrichment of the BY allele of *SSD1* in diploids. (A) Percentage of populations autodiploidized among the spores selected in SD –Ade –His +Can and SD –Ade –His –Ura –Trp +Can media after evolving for 500 generations. The numbers inside square brackets denote the number of populations in each category. Populations with ambiguous ploidy status are shown as haploids. (B) Percentage of sequencing reads at *SSD1* locus matching BY allele in haploid and diploid pools of the “selected spores.” Here *n* denotes the total number of reads at *SSD1* locus for each pool.

While the tetrad spores were well-suited to allow mapping of strong QTLs, we predicted that QTL inference might be hindered if the various combinations of auxotrophic markers, drug markers, and mating types in these spores affected autodiploidization. To hedge against this possibility, we also evolved 367 clonal *MAT***a** populations founded by unique “selected spores” from the same cross, bearing one of two sets of common auxotrophies (see Materials and Methods). Among these, 184 populations autodiploidized and 179 remained haploid, with 4 ambiguous (Figure 3A).

### *SSD1* drives differential autodiploidization propensity

To investigate the genetic basis of the difference in autodiploidization propensity between YAN463 and yGIL646, we sequenced each of the 260 F1 segregants in the tetrad set. We then conducted a standard QTL mapping analysis to identify associations between each SNP in the cross and the phenotype described above (specifically, whether the population founded by that segregant autodiploidized after 500 generations of laboratory evolution). We found a single strong QTL on chromosome IV (Figure 2C, LOD = 15.64, *P*** **<** **0.004). The second highest LOD score belonged to the *MKT1* locus on chromosome XIV, but this was not statistically significant (LOD = 2.64, *P*** **=** **0.13). These results remained unaltered when the above analysis was instead performed using the Haley–Knott regression method (Supplementary Figure S3).

To further evaluate whether any other QTLs played a significant role in determining this phenotype, we performed a test for multiple QTLs that allowed for interactions between loci. Using ∼58% of our populations as a test set, we employed a forward/backward stepwise search algorithm to develop a model that allowed for up to 4 interacting QTLs (see Materials and Methods for details). However, this search process ultimately found that a single-QTL model implicating the same chromosome IV locus performed best. This model also fits the held-out data (χ^2^ test, *P*** **< 10^-9^** **, *F* test, *P*** **< 10^-9^** **), yielding an overall LOD score of 8.63 and explaining 31.5% of the variance in the data.

To confirm this result, we performed a separate single-QTL analysis on the original dataset in which we regressed out the chromosome IV QTL. This analysis yielded no additional statistically significant QTLs (Supplementary Figure S4).

We found that the BY-allele of the chromosome IV QTL conferred a higher autodiploidization propensity (mean effect ± SE= 0.69 ± 0.04), while the W303-allele diminished autodiploidization in evolving populations (mean effect ± SE = 0.18 ± 0.04; Figure 2D).

To identify the specific gene underlying the significant chromosome IV QTL, we performed nucleotide BLAST (Madden 2013). This algorithm uniquely mapped the QTL to a single SNP in the *SSD1* gene. In BY, *SSD1* codes for a 1250aa-long mRNA-binding translational repressor. By contrast, the W303 *SSD1* allele (henceforth *ssd1-d*) harbors a G→C substitution resulting in a premature stop codon at the ORF’s 698^th^ codon (Y698*). This nonsense mutation effectively truncates the ORF by ∼44%.

To verify the findings of the tetrad experiment, we grouped the “selected spores” based on their ploidy and auxotrophy status (see Materials and Methods for details) and obtained metagenomic sequences of those pooled samples. Analyzing this data, we found that the proportion of reads matching the BY allele (*SSD1*) was substantially lower in haploid pools than in diploid pools (Figure 3B), irrespective of their auxotrophic status (Supplementary Figure S5). These results provide independent evidence that *SSD1* is the primary determinant of divergent autodiploidization propensity in clonal BY and W303 populations.

We observed slight but significant differences between the two sets of “selected spores” with respect to their auxotrophic genotypes. While proportions of diploids in both sets are close to 50%, populations founded by spores selected for the presence of both *URA3* and *TRP1* were slightly more likely to autodiploidize (χ^2^ test, *P*** **=** **0.0057, Figure 3A). This difference may be explained by the presence or absence of certain auxotrophic markers. For example, among the “tetrad spores,” we found that populations founded by spores prototrophic for tryptophan were marginally more likely to undergo autodiploidization (χ^2^ test, *P*** **=** **0.030), similar to the pattern observed among “selected spores” (Supplementary Table S1 and File S2; we find a similar effect in *LYS2* prototrophs but not for *URA3*). However, if *TRP1* (or a linked locus) does in fact have an effect on this phenotype, it is too small for our QTL analysis to detect.

### Populations with a functional copy of *SSD1* autodiploidize more frequently

To test the findings of the QTL mapping analysis described above, we used variants of *HygMX* and *KanMX* cassettes (see Materials and Methods) to construct BY4741 (BY) and yGIL104 (W303) strains in which their *SSD1* alleles had been either swapped or knocked out entirely, with appropriate controls. In total, we produced 3 strains on the BY background (BY4741, *ssd1*Δ; BY4741, *ssd1Δ::SSD1*; and BY4741, *ssd1Δ::ssd1-d*) and 3 on the W303 background (yGIL104, *ssd1*Δ; yGIL104, *ssd1Δ::SSD1*; and yGIL104, *ssd1Δ::ssd1-d*). Biological replicates of each strain were produced during the cloning process. Allele swaps were generated by knocking out *SSD1* with *HygMX* and re-introducing the appropriate allele with *KanMX*. Knockout strains were constructed by directly transforming *KanMX* into the *SSD1* locus or, as a control, by using *KanMX* to replace *HygMX* in the penultimate strains in the allele swap constructions.

We founded at least 22 haploid populations from each of these genotypes, divided among the available biological replicates. As in the previously described evolution experiment, we propagated these populations in rich media supplemented with ampicillin on 24-hour cycles, diluting 1024-fold each day and freezing portions of each population every 5 days.

**Figure 4:**
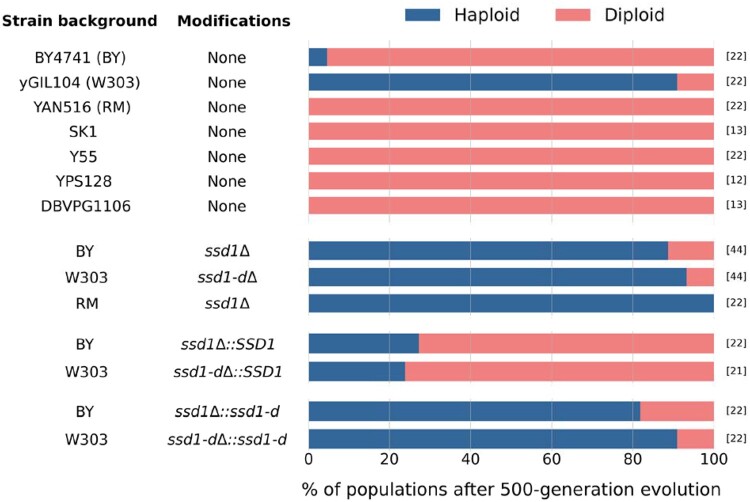
The effect of *SSD1* on autodiploidization. A nonfunctional *SSD1* gene reduced autodiploidization in W303 populations, while BY, RM, and other domesticated and wild strains expressing full length Ssd1 protein autodiploidized with high frequency. Knocking out *SSD1* reduced autodiploidization in BY and RM, making their frequency similar to that of W303. Allele swap experiments showed that irrespective of the genetic background, presence of the allele expressing the full length Ssd1 protein led to increased autodiploidization, whereas the allele expressing truncated Ssd1 protein reduced it. The numbers in square brackets denote the total number of clonal replicates for each strain. The full genotype of each strain can be found in Table 1.

As before, we found that almost all populations of the BY strain bearing its native *SSD1* allele autodiploidized during evolution in rich media for 500 generations (21/22, or 95%), while 20/22 (91%) populations founded by the W303 strain remained haploid. However, populations founded by either BY or W303 strains in which *SSD1* had been knocked out mostly remained haploid [39/44 (87%) and 41/44 (93%), respectively; Figure 4]. Similarly, populations founded by BY and W303 strains in which the native *SSD1* allele was replaced by the W303 *ssd1-d* allele also mostly remained haploid [18/22 (82%) and 20/22 (91%), respectively]. In contrast, populations founded by BY or W303 strains in which the native *SSD1* allele was replaced by the BY version of *SSD1* largely autodiploidize over the course of 500 generations of evolution [16/22 (73%) and 16/21 (76%), respectively; Figure 4]. Note that populations founded with BY strains in which *SSD1* was knocked out and reintroduced exhibited a marginally higher frequency of autodiploidization than populations founded with wild-type BY (binary logistic regression using IBM SPSS Statistics Software v26.0, Wald = 3.336, *P*** **=** **0.068) (IBM Corp 2019). While we do not know why this is the case, we suspect it may be due to changes in gene expression brought about by replacing the native terminator with a synthetic terminator, and/or by placing the *KanMX* gene immediately downstream.

To evaluate whether these results generalized to more distantly related *S. cerevisiae* strains, we also evolved 12 to 22 replicate populations founded by five other yeast strains [RM11-1a (RM), SK1, Y55, YPS128, and DBVPG1106] for 500 generations. Like BY, these strains all contain functional copies of *SSD1*, but represent two different allelic classes defined by amino acid differences at positions 1190 and 1196, in addition to three other variable sites (Supplementary Table S2; Cubillos *et al.* 2009; Cherry *et al.* 2012; Scopel *et al.* 2021). We find that all evolved populations diploidized over the course of evolution, regardless of their prototrophy for tryptophan (Figure 4 and Table 1). To understand whether *SSD1* played an important role in this phenotype for other strains, we constructed versions of RM in which the native *SSD1* was knocked out with *KanMX*, just as it was in BY and W303. We evolved 22 replicate populations founded with this knockout genotype (spread across two biological replicates) for 500 generations. We found that knocking out *SSD1* prevented autodiploidization in all replicates (Figure 4).

## Discussion

Ploidy changes mark a major shift in the biology of an organism, with potential consequences for the evolutionary dynamics of populations in which they occur. Although such ploidy changes have been seen frequently in natural, laboratory, and clinical settings, the genetic and environmental factors that influence these changes remain largely unknown. In this study, through experimental evolution and QTL mapping analysis, we find that the gene *SSD1* plays a central role in the emergence and fixation of diploids through spontaneous whole-genome duplication in evolving haploid yeast populations. Our results show that a fully functional *SSD1* gene enables population autodiploidization, whereas a complete knockout or hypomorphic variant of this gene [as observed in 7 of ∼1000 sequenced isolates (Peter *et al.* 2018, Scopel *et al.* 2021)] impedes it substantially.

Further work is needed to understand exactly how *SSD1* affects autodiploidization during experimental evolution. The Ssd1 protein is known to affect many important traits, such as aging, responses to stress, cell wall integrity, and bud formation (Kaeberlein and Guarente 2002; Kaeberlein *et al.* 2004; Kurischko *et al.* 2011; Li *et al.* 2013; Hu *et al.* 2018; Miles *et al.* 2019). This pleiotropic footprint makes it hard to speculate about the ultimate mechanisms responsible for *SSD1*’s effect on autodiploidization. For example, one recent study implicated *SSD1* in the maintenance of regular mitochondrial physiology and cytosolic proteostasis crucial for aneuploidy tolerance in wild yeast, showing that that W303 is sensitive to aneuploidy toxicity, which can be rescued with a functional copy of *SSD1* (Hose *et al.* 2020). Other recent work also provides evidence that yeast lacking *SSD1* are less tolerant of aneuploidies, and it seems this deficiency can be complemented by provision of either of two common functional *SSD1* alleles (Scopel *et al.* 2021). A similar mechanism may lead to reduced fitness for autodiploidized W303 cells as well, precluding their proliferation in the population. In addition, previous studies have shown that cell volume roughly doubles with doubling ploidy (Storchova 2014 and references therein). This may make proper *SSD1* function more critical in diploids than haploids, as it is a key regulator of cell wall growth and remodeling. Moreover, another recent study of budding yeast showed that *SSD1* facilitates entry, longevity, and recovery from cellular quiescence (Miles *et al.* 2019). W303 was shown to have diploid-specific defects in cellular quiescence and stationary phase viability that could be rescued by the introduction of a functional *SSD1*.

Together, these pieces of evidence suggest that a lack of functional Ssd1 protein in W303 cells may mediate the observed differences in population autodiploidization propensity by conferring a fitness disadvantage on autodiploids, independent of the frequency with which they occur de novo in the population. Of course, it is possible that *SSD1* also modulates the baseline per-division frequency of autodiploidization, or influences autodiploid fixation by other, more complex mechanisms (Gerstein and Otto 2011). Delineating these mechanisms is beyond the scope of the current study and a ripe area for future work.

In addition, while populations bearing *SSD1* knockouts or *ssd1-2* typically remained haploid over 500 generations of evolution in these experiments, an appreciable proportion did in fact autodiploidize (Figure 4). This suggests that, beyond the underlying per-division rate of diploidization and the relative fitness of newly minted diploids, dynamical factors such as clonal interference or the shifting distribution of fitness effects may also substantially influence the likelihood of autodiploid fixation. In addition, as indicated by our finding that *TRP1* (or linked loci) may also have a slight effect on this trait, it is possible that other loci besides *SSD1* play a role, and the mechanistic basis of their influence also remains to be determined. Further, although our findings point to a likely genetic explanation for differing frequencies of autodiploidization historically observed among yeast evolution experiments, it contrasts with the findings of Fisher *et al.* (2018), who observed autodiploids take over at high rates in adapting haploid W303 populations. Future work will be necessary to resolve this apparent discrepancy.

Finally, we note that the results here are limited since they only reflect evolution in a single rich media environment. Autodiploidization propensity has been reported to vary with environment (Harari *et al.* 2018), and it is possible the genetic basis of the trait may vary with environment as well.

In conclusion, we have shown that the frequency at which autodiploids take over adapting populations differs substantially between two closely related laboratory strains of *S. cerevisiae*. We have identified *SSD1* as the key genetic factor underlying the reduced autodiploidization in W303 compared to other strains. Using multiple laboratory and wild strains of *S. cerevisiae*, we showed that, irrespective of genetic background, strains with a functional copy of *SSD1* autodiploidize more frequently, while knocking out or truncating this gene reduces autodiploidization propensity. The results from this study suggest one strategy for modifying the frequency with which diploids take over experimental haploid budding yeast populations. In addition, we speculate that *SSD1* may be a potential target for modifying the rate of ploidy changes and genome stability in commercial settings, such as the large-scale production of economically important metabolites, and in clinical scenarios, such as the treatment of pathogenic fungal diseases and some cancers.
